# Mechanisims of asthma and allergic disease – 1089. The effect of the gender of the fetus on the control of asthma during pregnancy

**DOI:** 10.1186/1939-4551-6-S1-P85

**Published:** 2013-04-23

**Authors:** Mohamed Helmi Zidan, Hesham Adel

**Affiliations:** 1 Department of Chest Diseases, Alexandria Faculty of Medicine, Alexandria, Egypt; 2 Department of Obstetrics and Gynecology, Alexandria Faculy of Medicine, Alexandria, Egypt

## Background

Studies suggest that the presence of a female fetus worsen maternal asthma by up regulating maternal inflammatory pathways. Unlike the presence of a male fetus that produces a flow of testosterone during the second trimester which is considered a protective factor against asthma. The secretion of testosterone influences β-adrenergic-mediated relaxation of bronchial tissues and obstructs response to histamine, resulting in better improvement in asthma symptoms.

## Methods

476 pregnant women with physician-diagnosed asthma were enrolled during the first trimester of pregnancy and were followed up with a series of controlled maternal interviews throughout the pregnancy. The interviews assessed the level of asthma control using a 5-point scale. All medical records including information on hospitalization and unscheduled clinic visits for asthma since the last menstrual period were collected.

## Results

A higher proportion of women carrying a girl had poor asthma symptom control during the first part of pregnancy (24.1%) compared with asthmatic women carrying a boy (11.6%; *p* = 0*.*003). There was almost no difference noticed in the asthma symptom control during the second or third trimesters between women carrying a female fetus versus womwn carrying a male fetus. female-bearing pregnancy was associated with a significant increase in hospitalizations for asthma exacerbations (22.6%) compared with male-bearing pregnancy (8.8%; *p* = 0*.*01). Also the presence of a female fetus was associated with a higher incidence of unscheduled asthma visits during pregnancy (p=0.041).

## Conclusions

In conclusion, this study illustrated that the presence of a female fetus might increase asthma exacerbations during pregnancy compared to that of a male fetus. This evidence was supported by the number of unscheduled visits and hospitalizations in comparison to that in the case of a male fetus. However, the biological mechanism which could possibly defend the influence of fetal gender on the control of maternal asthma remains unstipulated. In this study we also noted the importance of early assessment and careful monitoring of all asthmatic pregnant women beside the major role of health education and environmental control when dealing with an asthmatic patient.

